# The regulatory landscape of interacting RNA and protein pools in cellular homeostasis and cancer

**DOI:** 10.1186/s40246-024-00678-6

**Published:** 2024-09-27

**Authors:** Carlos J. Gallardo-Dodd, Claudia Kutter

**Affiliations:** Department of Microbiology, Tumor, and Cell Biology, Science for Life Laboratory, Karolinska Institute, Solna, Sweden

**Keywords:** Noncoding RNA, RNA-binding protein, RNA modification, Post-transcriptional regulation, Cancer

## Abstract

Biological systems encompass intricate networks governed by RNA-protein interactions that play pivotal roles in cellular functions. RNA and proteins constituting 1.1% and 18% of the mammalian cell weight, respectively, orchestrate vital processes from genome organization to translation. To date, disentangling the functional fraction of the human genome has presented a major challenge, particularly for noncoding regions, yet recent discoveries have started to unveil a host of regulatory functions for noncoding RNAs (ncRNAs). While ncRNAs exist at different sizes, structures, degrees of evolutionary conservation and abundances within the cell, they partake in diverse roles either alone or in combination. However, certain ncRNA subtypes, including those that have been described or remain to be discovered, are poorly characterized given their heterogeneous nature. RNA activity is in most cases coordinated through interactions with RNA-binding proteins (RBPs). Extensive efforts are being made to accurately reconstruct RNA-RBP regulatory networks, which have provided unprecedented insight into cellular physiology and human disease. In this review, we provide a comprehensive view of RNAs and RBPs, focusing on how their interactions generate functional signals in living cells, particularly in the context of post-transcriptional regulatory processes and cancer.

## Background

Biological systems are entities of high complexity, with genomically encoded proteins and RNAs serving as key building blocks to give rise to functional cues. Both RNA and protein pools are major contributors to the assortment of biomolecules constituting the cell (1.1% and 18% of the total weight of mammalian cells, respectively) [1], and act in concert to govern virtually all cellular processes. Albeit different, RNAs and proteins assume essential structural, catalytic, and regulatory roles within the cell, modulating central processes all the way from genome organization and transcription to post-transcriptional regulation, subcellular localization, and translation [2, 3]. These events are often mediated by cooperative interactions between RNAs and RNA-binding proteins (RBPs), which in turn enable the assembly of effector ribonucleoprotein (RNP) complexes. Depending on the expression and location of RNAs and RBPs, RNPs can have shared or tissue-specific functions. Most importantly, the integrity of these intricate RNA-RBP networks is of utmost importance for maintaining cellular homeostasis, where perturbations affecting individual components can have profound effects on human pathophysiology, including the development of neurodegenerative disorders and cancer [4, 5].

For over a decade, the fraction of the nearly 3.1 billion base pairs in the human genome deemed functional has remained controversial. Notably, the concept of biological function is often understood as the capacity to contribute to cellular or organismal fitness. However, the attribution of function does not necessarily imply a reproductive advantage in all cases or may confer this advantage only under certain conditions. Estimates of the functional fraction of the human genome have ranged from as little as the protein-coding regions (~ 1–2%) to the full repertoire of genomic DNA covered with functional features determined by the Encyclopedia of DNA Elements (ENCODE) project (80%) [6–9]. Moreover, evolutionary-based efforts have attempted to estimate this fraction using sequence divergence or conservation information (3–15%) [10–13]. However, mapping the complete effective component of the human genome has proven challenging, especially in regions that are not actively transcribed or translated into functional protein products. Many of these genomic regions exhibit activity without clear patterns of evolutionary constraint at the sequence level. This has led to the investigation of additional dimensions to uncover conserved relationships of noncoding elements that can be indicative of function, including structure, spatiotemporal expression, and regulatory conservation (i.e., the maintenance of interactions and regulatory circuitry between distantly related species) [14]. In particular, noncoding RNAs (ncRNAs) are a diverse class of RNA molecules that originate from both genes (i.e., sense, antisense, intronic or gene overlapping) and intergenic regions of the genome and harbor unique potential to regulate central biological processes. The variable lengths of ncRNAs are accompanied by differences in sequence conservation. Shorter ncRNA genes tend to be more conserved, similar to protein-coding genes, whereas longer ncRNAs often exhibit evidence of rapid sequence evolution [15–17]. Nonetheless, there have been ever-growing reports of functional ncRNAs independent of their length and sequence conservation status [18–26]. A prominent feature is their ability to form complex RNA structures that facilitate interactions with RBPs. These structural elements are particularly predictable and measurable for small ncRNAs but have proven difficult to identify in long ncRNAs in which interspersed covarying sites are separated by longer stretches of nucleotide sequences. Moreover, it is even harder to define the cases where these structural elements are relevant in a biological context [27].

At the RNA-RBP interface, the protein’s composition and its ability to recognize RNA sequence and structural motifs are key aspects. Great progress has been made over the last decades in the RBP field, from initial discoveries revealing the existence of conserved RNA-binding domains (RBDs) to more recent observations that RBPs interact with RNA molecules via intrinsically disordered regions [28, 29]. In addition, the advent of high-throughput technologies has not only enabled the deep characterization of both protein and RNA pools within the cell but also allowed to capture RNA-RBP interactions at an unprecedented scale. Currently, the estimated number of RBPs extends well beyond those that contain canonical RBDs (up to 5,000 RBPs in humans) [30–33]. However, the corresponding RNA interactomes have been described for only a fraction of these proteins, and even fewer have well-defined functional roles. The ENCODE project has produced the most comprehensive resource to date, establishing RNA-RBP dependencies for a total of 150 RBPs in human cell lines [3]. Their approach combined experimentally derived interaction maps for RBPs with in vitro detection of binding specificities and functional assays, which highlighted the importance of using integrative approaches for elucidating functional RNA-RBP interactions. Besides ENCODE, additional databases have become valuable resources for exploring and analyzing RNA-RBP interactions. For instance, POSTAR3 [34] collects RBP annotations and interactions together with additional information layers from public resources to facilitate the exploration of post-transcriptional regulation mechanisms. Similarly, EuRBPDB [35] provides a multidimensional view of a comprehensive set of eukaryotic RBPs by combining data from various experimental observations. Databases such as starBase/ENCORI [36] or RNAInter [37] are specialized for RBP interactions with different RNA types, while others, including RBR-ID [38] and RBPDB [39], focus on RNA-binding regions within RBPs. Other repositories collecting RNA-RBP interaction data include RBPbase [5] and RBP2GO [40]. Alongside these efforts toward the systematic profiling of RNA-RBP associations, novel computational methods are being developed to facilitate the integration of orthogonal data types [24–26, 34, 41, 42]. Notably, reconstructing RNA-RBP regulatory networks is an essential step in the quest of understanding cellular homeostasis and treating disease.

In this review, we broadly present the functional landscape of interacting RNA and RBP pools in eukaryotic cells, focusing on the diversity of RNA species, their biogenesis, and central regulatory principles dictating RNA life. For an RBP-centric perspective, we refer readers to the following reviews [5, 28, 43, 44]. We highlight the emerging roles of ncRNAs as drivers of cellular phenotypes, emphasizing their implications in modulating protein synthesis in healthy and cancer cells. Lastly, we exemplify methodological advances empowering the study of RNA-RBP regulatory networks and their clinical relevance.

## RNA diversity and riboregulatory roles

**Fig. 1:**
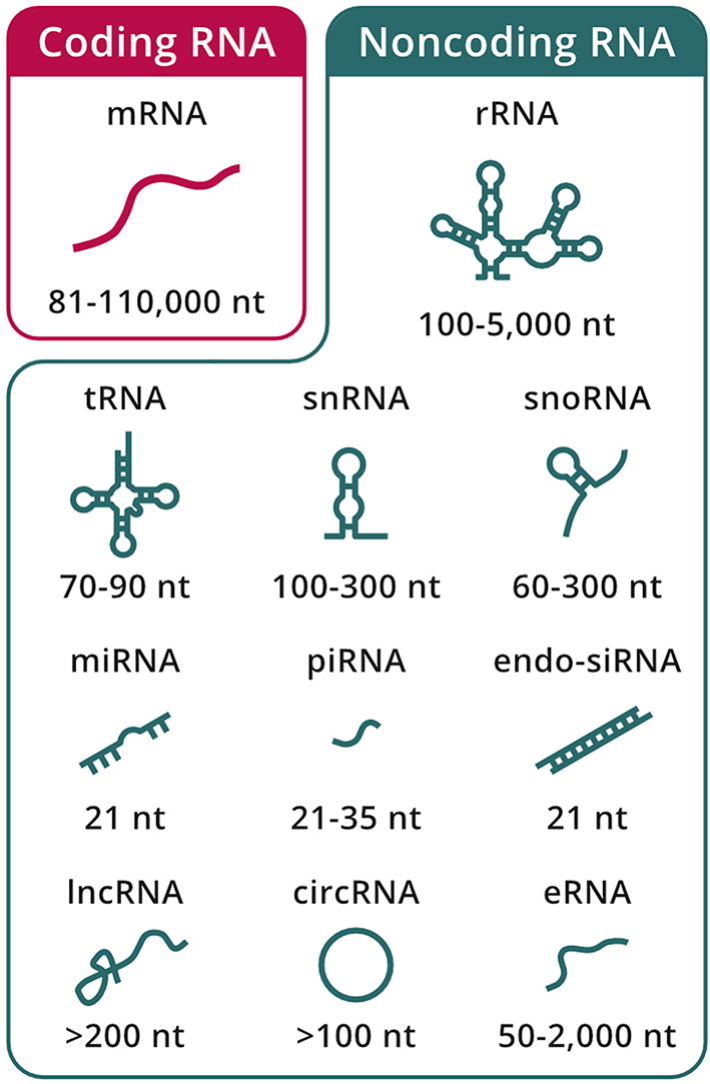
Diversity of the eukaryotic transcriptome. RNA species are separated into coding (red box) and noncoding (green box) classes. The approximate length of each individual RNA type is indicated.

High-throughput technologies have revolutionized the world of RNA biology, changing the view of RNA molecules from just carriers of genetic information to a diverse functional class orchestrating a wide array of processes in living cells [45, 46]. Current understanding separates messenger RNAs (mRNAs), which have protein-coding potential, from ncRNA species that exert a variety of roles independent of the ability to generate a functional protein product (Fig. 1). Several attempts have been made at categorizing ncRNA types, but the most evident grouping distinguishes only between long ncRNAs, for those greater than 200 nucleotides in length, and small ncRNA, for those below this arbitrary cutoff [47]. However, there are still certain ncRNA species that are not clearly classified by this criterion, with examples both above and below the 200-nucleotide threshold. Alternatively, ncRNAs can be grouped by function into constitutive and regulatory types [48] but also stratified based on their biogenesis pathways, including differences in transcription caused by dedicated DNA-dependent RNA polymerases (Pols) [49, 50].

**Fig. 2:**
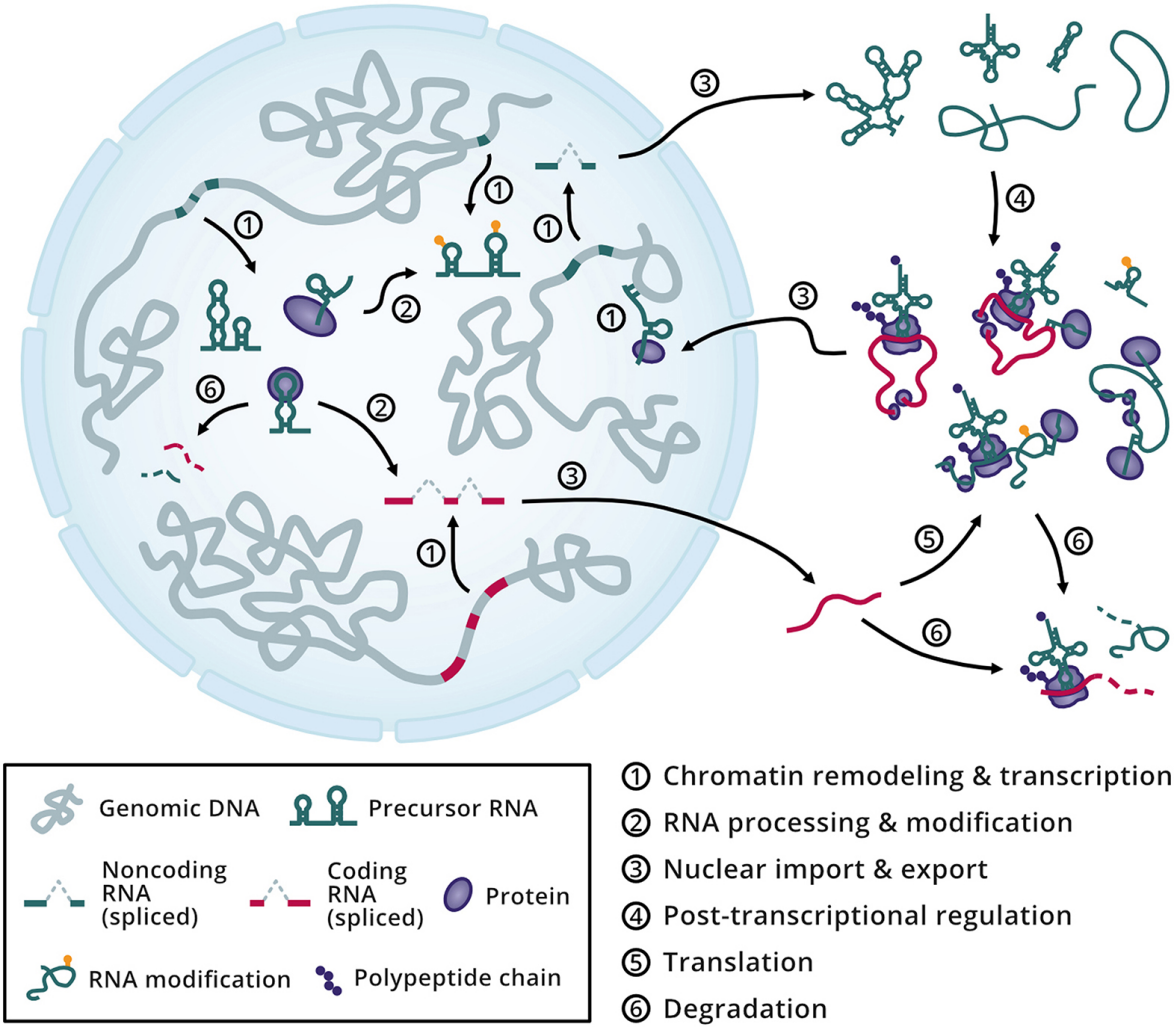
Overview of transcriptional and post-transcriptional processes influenced by RNA-RBP interactions. In the nucleus (blue sphere), nascent RNAs are actively processed and modified before being exported to the cytoplasm (white), where additional processing follows. In the cytoplasm, messenger RNAs (red lines) are translated into proteins (purple shapes). Noncoding RNAs (green lines) interface with the protein pool to shape post-transcriptional regulatory processes in the cytoplasm or translocate to the nucleus as ribonucleoprotein complexes to remodel gene expression programs. Decay of molecules occurs simultaneously, with degradation determining turnover rates in the cell.

During and after transcription, binding events between RNAs and RBPs are fundamental for regulating most cellular processes (Fig. 2), shaping the fate of not only the individual molecules but also that of assembled RNP complexes and the cell itself. Nascent RNAs undergo a series of co-transcriptional processing events. These steps involve the RBP-mediated removal of sequences from the nascent RNA molecule, which is often referred to as primary or precursor RNA (pre-RNA). Additionally, RBPs can chemically modify nucleotides within RNA molecules. Further RBP-controlled RNA processing occurs within the nucleus or in the cytoplasm after export through nuclear pore complexes (NPCs), resulting in mature transcripts. These and subsequent steps are collectively referred to as post-transcriptional regulation of gene expression. At this stage, RBPs continue to interact with RNAs within the cellular environment, defining core processes such as translation and degradation, and ultimately dictating turnover rates and cell functioning (Fig. 2). Moreover, newly synthesized proteins and RNAs can shuttle back and forth between the nucleus and cytoplasm to exert their different roles. For instance, RBPs can translocate to the nucleus to guide chromatin remodeling through RNA-binding and change gene expression programs. An example of this involves the ncRNA *HOTTIP*, which interacts with the WDR5/MLL remodeling complex to promote histone 3 lysine 4 trimethylation (H3K4me3) of genes at the *HOXA* locus, enhancing their expression [51]. However, other examples are highly discussed, such as the case of X chromosome inactivation in mammals, where the ncRNA *X-inactive specific transcript* (*XIST*) recruits, among other factors, the Polycomb repressive complex 2 (PRC2) to establish a H3K27me3-repressive chromatin state that silences one copy of the X chromosome in females [52–55].

There is a great diversity of RNA species, each of which participates in different cell functions and has specific RBP interaction partners. In the following sections, we briefly introduce the major RNA types together with their roles and discuss the core processes for cellular homeostasis that are driven by RNA-RBP specificities.

### Transcriptome heterogeneity

The cell harbors an ample array of RNA molecules that come in different shapes and sizes (Fig. 1). These differences are also reflected in terms of their biogenesis pathways, abundance, and roles within the cell [45, 48, 50, 56].

#### Messenger RNA (mRNA)

Among all RNA types, mRNAs (~ 8,900 molecules per million) carry the information needed for protein production. Transcribed by eukaryotic Pol II, mRNA molecules are subjected to multiple processing steps including capping of 5’ ends, splicing, cleavage, and polyadenylation at the 3’ end [57]. Adequate processing of these RNAs into mature mRNAs is essential for translation in eukaryotes, as this enables the recognition by translation initiation factors, ribosome assembly, and protein synthesis [58]. The size of mRNAs varies greatly from as little as 81 to approximately 110,000 nucleotides for the longest protein annotated in humans, which is encoded by the *TTN* gene [59].

#### Ribosomal RNA (rRNA)

The ribosome is a remarkable RNP complex that translates mRNAs into proteins. It is largely composed of highly abundant rRNAs (~ 89,000 molecules per million), which form the large (60 S: 28 S, 5 S and 5.8 S rRNAs) and small (40 S: 18 S rRNA) subunits together with ribosomal proteins [60]. The transcription of rRNA genes is driven by Pol I (45 S rRNA) and Pol III (5 S rRNA) and localizes to the nucleolus and its vicinity, a highly active region in the nucleus that acts as a hub for the cleavage and modification of ribosomal components [61–63]. For instance, the 45 S pre-rRNA is further processed into mature 28 S, 18 S and 5.8 S rRNAs for ribosome assembly [64]. Their mature length can range from around one hundred to several thousand nucleotides.

#### Transfer RNA (tRNA)

Like rRNAs, tRNAs (~ 890,000 molecules per million) are highly abundant, modified and structured ncRNA molecules that enable protein synthesis. They function by complementarily binding nucleotide triplets (codons) at the ribosome-mRNA interface and adding their charged amino acids to the growing polypeptide chain [65]. There are a total of 429 highly confident tRNA genes annotated in the human genome, which are transcribed by Pol III and can be classified into 47 isoacceptor groups according to their anticodon sequence [66–68]. After processing, their length is approximately 70 to 90 nucleotides. Notably, mature tRNAs can be further processed by RBPs into tRNA-derived halves or tRNA-derived fragments (tRFs), which exert roles beyond mRNA translation, including the impairment of reverse transcription and silencing of endogenous retroviruses, or the displacement of stabilizing RBPs [69–71].

#### Small nuclear RNA (snRNA)

A characteristic of eukaryotic organisms is the presence of introns in pre-RNAs. These are accurately removed co- or post-transcriptionally by the spliceosome, a nuclear-localized RNP complex containing snRNAs (~ 4,100 molecules per million). Except for selected cases like the nuclear-processed U6 snRNA that is Pol III-transcribed [67], snRNAs are transcribed by Pol II and partake in the major (U1, U2, U4, U5 and U6 snRNAs) and minor (U5, U11, U12, U4atac and U6atac snRNAs) spliceosomal complexes that target divergent sets of intron sequences [72, 73]. Briefly, the processing of snRNAs involves 5’-end capping and 3’-end cleavage, followed by export to the cytoplasm for snRNP assembly and translocation back to the nucleus [72]. Moreover, snRNPs concentrate in nuclear substructures termed Cajal bodies, which are considered hubs for snRNP processing and remodeling. Their length varies in the range of 100 to 300 nucleotides.

#### Small nucleolar RNA (snoRNA)

The established role of snoRNAs (~ 3,400 molecules per million) is to guide RNA editing proteins to their target sites within other RNA sequences, for example rRNAs, snRNAs, and tRNAs. In addition, other roles have been defined for this class including the modulation of alternative splicing events or 3’ end processing of RNAs influencing transcript stability and abundance [74]. There are over 2,000 snoRNAs annotated in humans [75] that are transcribed by Pol II and separated into two groups according to their conserved sequence elements and interaction partners (i.e., C/D- and H/ACA-box snoRNAs). Box C/D snoRNPs classically mediate 2’-O-methylation, while box H/ACA snoRNPs mediate pseudouridylation of targets [74, 76]. In addition, certain snoRNAs from both C/D- and H/ACA-box types, which contain distinct features, such as CAB boxes (UGAG) and GU repeats, localize to Cajal bodies, where they facilitate snRNA processing. These are hence termed small Cajal body-specific RNAs. After transcription, snoRNAs are primarily released from intronic sequences of host genes in humans and are subsequently processed by cleavage at the 5’ and 3’ ends. This process results in a mature length of approximately 60 to 300 nucleotides.

#### MicroRNA (miRNA), PIWI-interacting RNA (piRNA) and small interfering endogenous RNA (endo-siRNA)

Silencing of gene expression mediated by miRNAs (~ 2,700 molecules per million) has been studied exhaustively since the original discovery of *lin-4* in *C. elegans* [77]. Like the other small ncRNA types described above, miRNAs act by guiding proteins to target RNA sequences through antisense complementarity. In this case, Argonaute (AGO)-bound miRNAs commonly enable the recognition of target sites toward the 3’ end of transcripts, leading to RNA degradation or translational repression [78]. Newly Pol II-transcribed miRNAs, termed pri-miRNAs, are processed by RNase III-type endonucleases of the Microprocessor complex (Drosha and DGCR8) into individual stem-loops, which are hairpin-like RNA structures that are essential intermediates in miRNA biogenesis and are also referred to as pre-miRNAs. Pre-miRNAs are then exported from the nucleus by exportin 5, followed by stem-loop cleavage by Dicer and AGO coupling as mature single-stranded miRNAs of 21 nucleotides in length [79]. Complementing miRNAs, small interfering RNAs that are produced endogenously (i.e., endo-siRNAs), such as those found in mouse stem cells, can be loaded onto AGO proteins to serve as guides for gene silencing. These endo-siRNAs are approximately 21 nucleotides in length [79, 80]. Moreover, piRNAs, which are primarily expressed in germline cells, interact with PIWI-AGO proteins to repress transposable elements, regulate gene expression, and fight viral infection [81]. These small ncRNA molecules are between 21 and 35 nucleotides in length.

#### Long noncoding RNA (lncRNA), circular RNA (circRNA) and enhancer RNA (eRNA)

Large-scale profiling of the human transcriptome has resulted in the identification of a substantial catalog of lncRNAs (~ 2,100 molecules per million), with recent annotations of the human genome setting their number on par with that of protein-coding genes at over 19,000 [82]. However, due to factors such as their low abundance and sequence conservation as well as restricted expression in specific cell types and under certain conditions, the detection and functional characterization of lncRNAs has remained a challenge [17]. Among all ncRNA types, lncRNAs exhibit the highest functional versatility, covering roles ranging from chromatin remodeling to transcriptional and post-transcriptional regulation [83, 84]. Similar to mRNAs, the transcription and processing of lncRNAs occurs via Pol II, including 5’-end capping, splicing, and 3’-end polyadenylation. A distinctive feature of mRNAs and lncRNAs is that the splicing process appears more inefficient in the latter, which affects their alternative splicing landscape [83, 85]. Moreover, there are other ncRNA molecules that are generally grouped with lncRNAs. For instance, circRNAs, which are covalently closed RNA molecules generated by back-splicing that are enriched in brain tissue, have been shown to modulate cellular processes by acting as miRNA sponges (i.e., molecules that sequester and inhibit miRNAs by binding to them, thereby preventing miRNAs from interacting with their target mRNAs), transporters, or scaffolds for RBPs [86, 87]. In addition, eRNAs, which are transcribed from active enhancer regions in the genome, have been shown to sustain enhancer activation and the expression of target genes [88, 89]. lncRNAs are usually defined as RNA molecules greater than 200 nucleotides in length although certain circRNAs and eRNAs may be shorter than this cutoff.

### RNA-RBP specificities guiding cellular processes

**Fig. 3:**
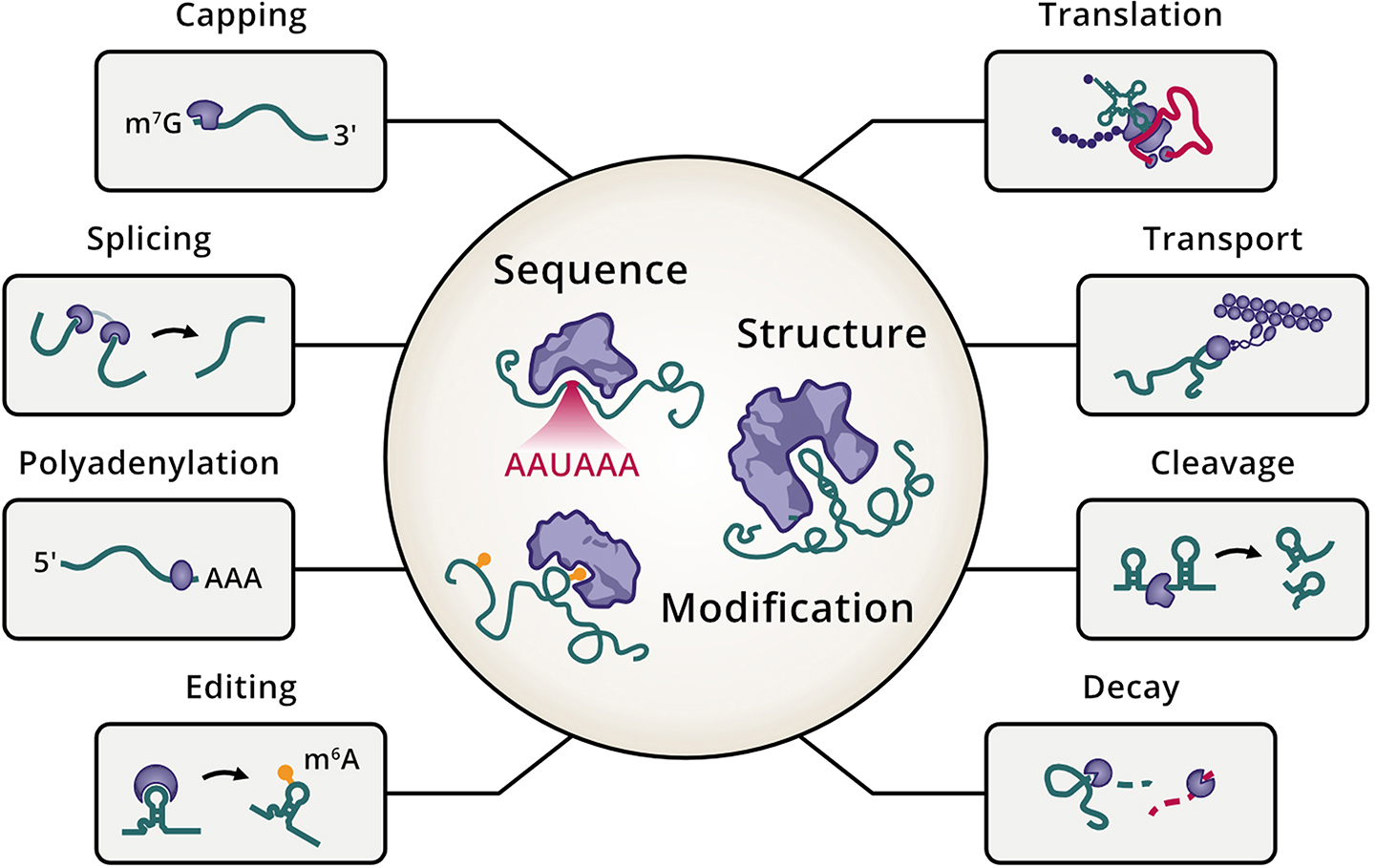
Determinants of RNA-RBP specificities and their functional implications. The RNA-RBP interface (central sphere) is facilitated by sequence elements, structural arrangements, and chemical modifications of RNAs (green lines) that enable recognition by selected proteins (purple shapes). RNA-RBP interactions intervene in multiple cellular processes. *Anticlockwise*: capping to add a m^7^G-cap protecting the 5’ end of RNAs, splicing to excise sequences from RNA molecules, polyadenylation to extend the 3’ end of RNAs with adenine stretches, editing to introduce chemical modifications (orange lollipops) in RNAs, decay to degrade RNA molecules, cleavage to cut RNAs, transport to localize RNAs and other molecules at different cellular locations, and translation to decode messenger RNA to produce proteins.

Many functional outcomes in the cell are governed by the specific recognition and coupling between RNA molecules and RBPs. These interactions rely on distinct sequence motifs, secondary structures, and post-transcriptional modifications at the RNA level that collectively orchestrate various cellular processes (Fig. 3). On the protein side, RBPs that utilize established RBDs to interact with RNAs are termed canonical RBPs. There is a wide variety of RBDs, including the more abundant RNA recognition motifs and K homology domains, but also less prevalent ones, such as those found in ribosomal proteins [31]. RBDs often repeat or co-occur with other RBDs, and the presence of multiple RBDs within a single RBP can greatly enhance the versatility and robustness of RNA interactions. In turn, RBPs lacking such domains are referred to as noncanonical, unconventional, or moonlighting RBPs given their alternative classical roles independent of RNA-binding [28]. The binding of noncanonical RBPs to RNA is facilitated via intrinsically disordered regions. Moreover, post-translational modifications such as phosphorylation or acetylation play crucial roles in modulating RNA-binding affinity and specificity. These modifications can alter the conformation of RBPs, influence their interaction with other proteins and cofactors, and regulate their localization and stability, thereby fine-tuning the cellular functions they govern. For instance, allosteric regulation via interdomain communication of tandem RNA recognition motifs has been shown to influence the ability of hnRNPA1 to interact with protein partners and bind RNA [90].

Coinciding with the switch from transcription initiation to elongation of Pol II-transcribed mRNAs and lncRNAs, the carboxy-terminal domain of Pol II is phosphorylated and recruits capping factors [91]. Subsequent enzymatic triphosphatase, guanylyltransferase, and methyltransferase activities result in the formation of a 7-methylguanosine cap (m^7^G-cap) structure at the 5’ end of the nascent RNA molecule, which is further bound by the cap-binding complex (CBC), mediating, among other processes, export from the nucleus [92]. Notably, the Pol III-transcribed 7SK snRNA is known to modulate the kinase activity needed for transcription elongation by sequestering the positive transcription elongation factor b (P-TEFb) [93]. Here, a combination of structural elements and sequence motifs enables the assembly of the 7SK RNP complex, which contains the HEXIM, LARP7 and MePCE proteins, and controls the repression of P-TEFb activity [94].

During the elongation phase of transcription by Pol II, intron sequences within the growing pre-RNA are removed by splicing. The spliceosome is built through the coordinated assembly of U-rich snRNP complexes that enable the recognition of key sequence elements defining intron boundaries. These are the GU 5’ (donor) and AG 3’ (acceptor) splice sites and the branchpoint sequence neighboring the splice acceptor (~ 18–40 nucleotides upstream) [95]. Successful splicing is accomplished after two subsequent transesterification reactions that occur co-transcriptionally as the transcript emerges from Pol II [95, 96]. Moreover, splicing regulatory elements (SREs) can influence the splicing process by recruiting trans-acting RNPs [97, 98]. Simultaneously during transcription, RNA editing factors can introduce (writers) or remove (erasers) chemical modifications at individual nucleotides harboring great potential for regulation [99, 100]. For instance, N^6^-methyladenosine (m^6^A) modifications can be recognized by the canonical RBP hnRNPG (reader), which has been shown to promote alternative splicing when they occur close to splice sites [101].

Such as with splicing, there are further processing steps of the pre-RNA that take place before transcription termination. This includes initiating polyadenylation (poly(A)) at the 3’ end by a poly(A) polymerase (PAP). The choice of poly(A) site is mediated by a core set of factors (CPSF, CSTF, CFI and CFII) that recognize the AAUAAA poly(A) signal together with auxiliary sequence elements and cleave the RNA molecule in the presence of PAP, resulting in the addition of a 3’ poly(A) tail of approximately 250 nucleotides [102–104]. Poly(A)-binding proteins (PABPs) that are bound during PAP synthesis have been shown to control poly(A) tail length [105]. Moreover, the differential usage of regulatory elements leads to alternative cleavage and poly(A) events that change 3’ untranslated regions (UTRs), influencing future steps of RNA life and adding to the diversity of the transcriptome [102, 104].

After these co-transcriptional processes, which are primarily but not exclusively linked to protein-coding RNAs, transcripts are exposed to additional post-transcriptional regulatory cues, including those aforementioned, that affect their stability, subcellular localization, or translation. Mature mRNAs are composed of both protein-coding and noncoding exonic regions that are retained after splicing. In fact, only a fraction of exonic sequences are annotated as protein-coding in eukaryotic genomes (~ 23% in humans), although noncoding regions of protein-coding RNAs have important regulatory roles mediated via interactions with RBPs and are often altered in disease [106]. For instance, RNA sequences and structures at the 5’ UTRs of mRNAs modulate ribosome recruitment controlling translation efficiency, and the use of alternative 5’ UTR isoforms has been shown to correlate with enhanced protein synthesis rates in diseases such as squamous cell carcinoma [107, 108]. Moreover, RNA elements at 3’ UTRs and poly(A)-tail length have been shown to influence mRNA stability, translation efficiency, and protein localization at different developmental stages [109, 110]. In the case of ncRNAs, they are assembled from noncoding exons (i.e., intergenic ncRNA genes) but can also arise from exons and introns of protein-coding genes through alternative transcription or pre-RNA processing. For example, the lncRNA *FAST* partially overlaps and is transcribed in the antisense direction of the *FOXD3* gene, and its expression has been shown to maintain pluripotency in human embryonic stem cells through the activation of WNT signaling [111]. In addition, the lncRNA-*PNUTS* is an alternatively spliced isoform generated from the protein-coding gene *PNUTS* that has been associated with breast cancer progression [112]. Importantly, the generalizability of regulatory cues exerted by RNA-RBP interactions is still debated since these are often context-dependent. Some level of contradiction in the literature can be explained by contrasting cellular states at different developmental stages (i.e., differentiating and differentiated cells) or in diseases such as cancer (i.e., dedifferentiating cells), where there are differences in the subcellular localization of RNA and RBP partners or changes in their binding affinities.

For export from the nucleus, mature transcripts interact with adaptor molecules and transport factors that facilitate their transit through the NPC. For example, transcription-export complexes (TREXs) bind mRNAs co-transcriptionally and are further recognized by the NXF1-p15 heterodimer, which mediates the export of mature molecules across the NPC [113]. Nonetheless, a detailed picture of the underlying mechanisms and RBP complements enabling the selective export of mature mRNAs and the array of other processed RNA species is still lacking.

In the cytoplasm, the CBC-bound m^7^G-cap structure of mRNAs binds to eukaryotic translation initiation factor 4E (eIF4E), which promotes protein production by recruiting additional translation initiation factors and circularizing RNA through interactions with PABPs anchored at the poly(A) tail [92, 114]. Among the different factors influencing translation, the availability of correctly processed, modified, and structured core molecules such as rRNAs and tRNAs is essential. For instance, codon bias and tRNA supply have been shown to ensure translation efficiency within the cell [115–117].

Furthermore, RNA decay pathways compromise the stability of mRNA molecules, leading to degradation and limiting the rate of translation. The processes contributing to degradation include deadenylation-dependent, deadenylation-independent, and endonuclease-mediated mRNA decay pathways [118]. Aside from their role in translational repression, miRNAs or siRNAs targeting the 3’ UTR of mRNAs can unfold mRNA decay by recruiting deadenylation (CCR4-NOT and PAN2-PAN3) as well as decapping (DCP1 and DCP2) factors, which enables degradation by the exosome complex (3’ to 5’) and exoribonuclease XRN1 (5’ to 3’), respectively [119, 120]. Moreover, both miRNAs and siRNAs can promote decay via cleavage at the target site when the sequence complementarity is high [121]. Surveillance mechanisms also occur in both the nucleus and the cytoplasm to ensure the integrity of transcripts [118]. For instance, nonsense-mediated decay recognizes and degrades mRNAs with premature termination codons that could result in truncated proteins. In addition, alternative translation-dependent mRNA decay mechanisms include the degradation of transcripts lacking a stop codon (non-stop decay) or with stalled ribosomes (no-go decay). Aberrant transcription or processing of mRNAs in the nucleus can lead to rapid degradation through homologous exosome and exoribonuclease activities to that of the cytoplasm.

Lastly, RNA transport is another key process facilitating the localization and formation of dense RNP complexes in different cellular compartments. Examples include canonical RBPs containing a YTH domain that selectively bind m^6^A-modified transcripts to localize at processing bodies (P-bodies) together with proteins associated with RNA decay [122] and zipcode-binding proteins (ZBPs), such as ZBP1, which localizes *Actb* mRNAs to the leading edge of migrating fibroblasts for translation [123]. Besides P-bodies, which function in mRNA degradation, storage and translational repression, there are other interesting subcellular condensates of phase-separated RNAs and proteins such as germ granules, neuronal granules, and stress granules [124, 125]. Germ granules are unique RNA-protein aggregates present in germline cells that contain piRNAs and AGO family proteins. They play a crucial role in modulating transcript stability and translation during gametogenesis and ensuring genome integrity through the silencing of transposable elements. Neuronal granules are composed of RBPs (e.g., FMRP, STAU2, TDP-43) that facilitate long-distance RNA transport in neurons and local translation at synapses. Stress granules are temporary assemblies of mRNAs and associated proteins, including G3BP1/2, TIA-1 and TIAR, that stall translation in response to cellular stress, helping to regulate gene expression under adverse conditions.

Overall, understanding the specificity and complexity of RNA-RBP interactions at the molecular scale provides unique insight into the elaborate regulatory networks that sustain cellular homeostasis. In the next section, we cover examples of RNA-RBP interactions influencing cellular phenotypes with a primary focus on the divergent roles of different ncRNA species in cancer.

## Mechanisms in cellular homeostasis and cancer

Given the central role of RNA-RBP interactions in cell function, it is not surprising that there is mounting evidence that perturbations in RNA-RBP regulatory networks lead to disrupted cellular physiology and human diseases, such as cancer. Aberrant gene expression and transcript formation are common in cancerous cells and disrupt self-renewal, proliferation, and differentiation processes, which are essential for maintaining cell homeostasis. Efforts to profile the expression and mutation rate of RBPs across human cancers have revealed substantial alterations that are potentially implicated in their cellular phenotypes [126, 127]. In fact, RBP signatures have been proposed as prognostic markers for hepatocellular carcinoma (HCC), underscoring the relevance of these genes [128]. Developmental studies have also highlighted the importance of RBPs during embryogenesis, whereby resuming RBP activity may result in oncogenic effects that enhance cancer traits [100, 129, 130]. Notably, these alterations are tightly associated with changes in RNA modifications, structure, and abundance, which in turn contribute to influencing the cellular processes driving the cancer phenotype.

**Fig. 4:**
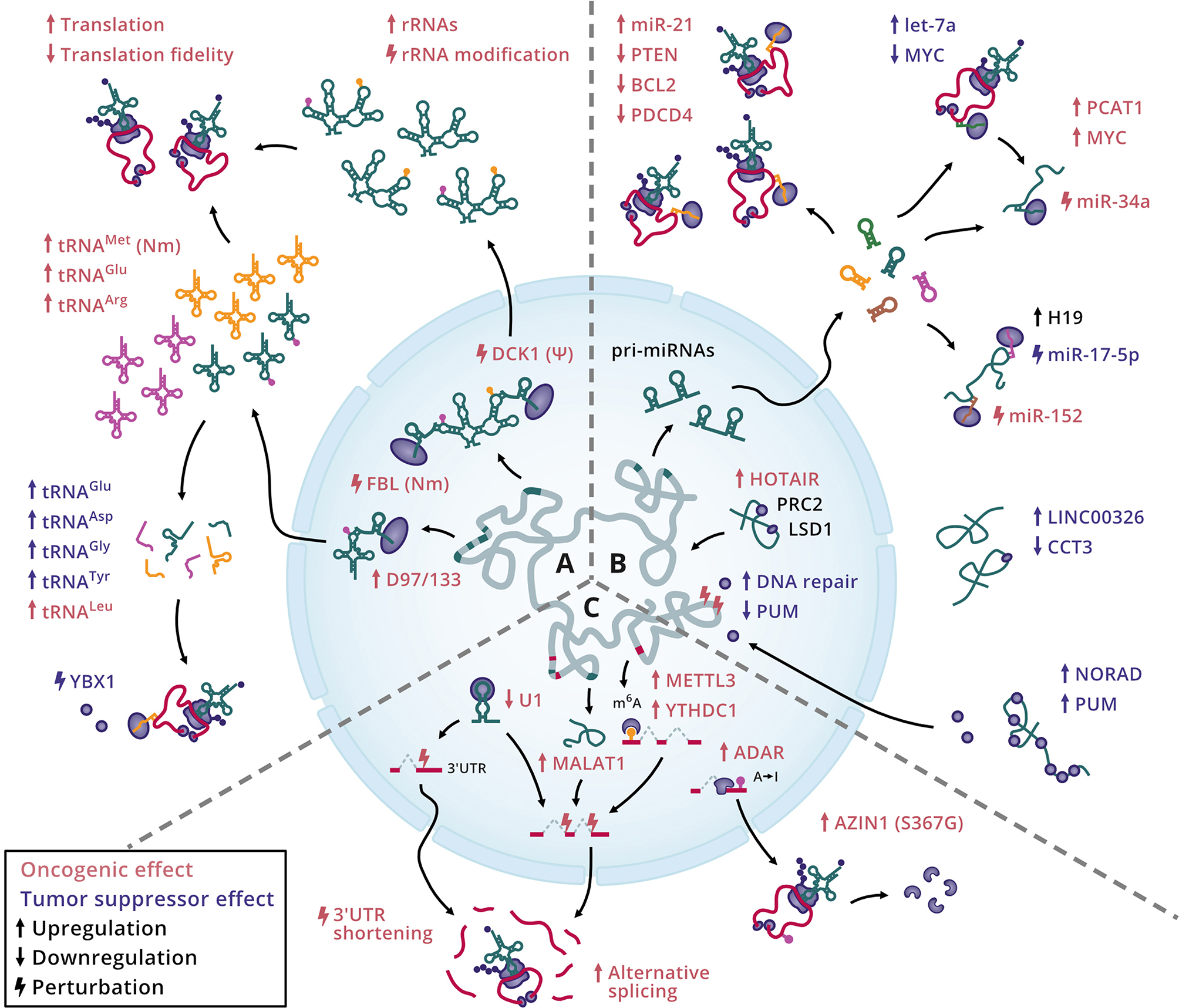
Cellular processes with oncogenic and tumor-suppressive roles involving RNA-RBP interactions. **A**) Alterations affecting the central components of the translation machinery. **B**) Regulatory processes mediated by noncoding RNAs that influence gene silencing and genome stability. **C**) Changes perturbing RNA modifications, the splicing process, and protein products.

The equilibrium of core components of the translation machinery is a fundamental aspect that defines cellular states. Consequently, disturbances in the rRNA pool and tRNA supply in the cell can perturb translation dynamics and promote cancer (Fig. 4A). Elevated proliferation in tumorigenesis requires efficient protein synthesis; therefore, the translation process is frequently dysregulated in cancer cells [131]. Several studies investigating the coupling of mRNA codons and tRNA anticodons in homeostasis have found that the translation efficiency landscape is stable overall across most mammalian cell types and during development [16, 132, 133]. The balance of codons and anticodons is further known to mediate translation elongation rates and mRNA stability [134]. However, changes to selected tRNAs have been shown to increase proliferative phenotypes and metastasis, such as the overexpression of tRNA^Glu^ (UUC) and tRNA^Arg^ (CCG) in breast cancer [135, 136]. In addition, increased rRNA levels have been associated with prostate and cervical cancer but are not always linked to promoter hypomethylation [137, 138]. Both rRNAs and tRNAs are extensively modified to fulfill their canonical roles, and changes in these modification patterns can also lead to the development of cancer. For instance, defective rRNA pseudouridylation (Ψ) mediated by snoRNA-guided Dyskerin (DCK1) alters rRNA and ribosomal structures, thus limiting the fidelity of translation and increasing cancer susceptibility [139]. Similarly, defects in rRNA 2’-O-methylation (Nm) by snoRNA-guided Fibrillarin (FBL) can compromise translation influencing cancer progression [140]. Indeed, recent work suggests that higher levels of SNORD97/133 may facilitate methionine-rich proliferation-related gene expression programs by increasing 2-O-methylation of target methionine tRNAs in cancer cells [141]. Besides this, tRFs, which were originally thought of as byproducts of tRNA degradation have been described as functional entities. Certain tRFs derived from tRNA^Glu^, tRNA^Asp^, tRNA^Gly^, and tRNA^Tyr^ have been shown to post-transcriptionally suppress oncogenic factors in breast cancer by displacing YBX1 binding at 3’ UTRs [69]. Conversely, tRNA^Leu^-derived tRFs can exert oncogenic roles by promoting the translation of selected ribosomal mRNAs [142].

An alternative regulatory layer in cancer biology encompasses the role of miRNAs and lncRNAs. The primary functions of miRNAs include mRNA destabilization and translational repression of oncogenes (oncomiRs) and tumor suppressors, whereas lncRNAs function through various modes of action including miRNA or RBP decoy activity (or sponging) and scaffolding to guide RNP complex assembly (Fig. 4B). Studying the roles of miRNAs in cancer is an area of intense research. For example, the oncomiR miR-21 is overexpressed in HCC, where it targets *PTEN* mRNA and inhibits its tumor suppressor activity, enhancing cell proliferation, migration, and invasion [143]. High levels of miR-21 are known to be associated with additional cancer types and downregulation of target genes, such as BCL2 and PDCD4, which mediate cellular apoptosis [144]. Moreover, miRNAs can also target oncogenes to exert tumor-suppressive effects. This applies to let-7a, which has been shown to reduce MYC levels, limiting cell growth in Burkitt lymphoma [145]. In the case of lncRNAs, they remain an enigmatic class of ncRNA, though a versatile set of functions impacting cancer progression has been described to date. Reports have shown that the lncRNA *H19* competitively binds to miR-17-5p and miR-152, acting as an endogenous sponge that diverts these miRNAs from their respective targets, YES1 and DNMT1, thus modulating cellular responses in thyroid and breast cancer, respectively [146, 147]. The lncRNA *PCAT1* exhibits an oncogenic role in prostate cancer by post-transcriptionally inducing *MYC* expression and safeguarding MYC levels from miR-34a-induced repression [148]. In addition, certain lncRNAs display protective effects against cancer transformation. For instance, *NORAD* can sequester PUM proteins and impede their nuclear function promoting genome instability [149]. Also, in the nucleus, *HOTAIR* acts as a lncRNA scaffold bridging PRC2 and LSD1 protein complexes to repress gene transcription and promote metastasis [150, 151]. Furthermore, the interaction between the chaperone and the noncanonical RBP CCT3 and *LINC00326* has been shown to regulate cellular metabolism, whereby downregulation of CCT3 enhances *LINC00326* expression to repress lipid accumulation and cell proliferation in HCC [23].

Notably, perturbations in the alternative splicing landscape can significantly impact the development of cancer. This can be influenced by changes in the splicing machinery, such as the availability of snRNAs and other splicing factors, as well as by RNA modifications altering key regulatory sequences in pre-RNA molecules (Fig. 4C). The U1 snRNA silences proximal poly(A) signals at the end of transcripts; hence, its downregulation has been shown to favor the shortening of 3’ UTRs as well as to alter the splicing and expression of cancer-related genes increasing cell migration and invasiveness [152]. Moreover, the lncRNA *MALAT1* has been associated with diverse roles in tumorigenesis and is known to regulate alternative splicing by influencing the levels of trans-acting splicing factors, such as serine/arginine-rich (SR) proteins [153]. Regarding RNA editing, an adenosine-to-inosine (A-to-I) modification of *AZIN1* transcripts mediated by ADAR proteins leads to a serine-to-glycine substitution at residue 367 (S367G), altering the AZIN1 structure and enhancing its activity, which drives pathogenesis in HCC [154]. Lastly, recent reports have implicated m^6^A modification readers, including METTL3 and YTHDC1, in the recruitment of splicing factors leading to different alternative splicing programs that can potentially explain tumor-associated phenotypes via the production of malignant proteoforms [155, 156].

## Conclusion

The ever-growing collection of dependencies between RNA and protein pools in living cells has contributed greatly to our understanding of molecular processes orchestrating cellular physiology in homeostasis and disease. Despite recent advances, our knowledge of RNA-RBP interactions remains limited and is constrained by the current catalog of experimental methods and computational tools. This is especially true for functionally diverse classes such as lncRNAs, where the mechanistic basis for recognition is highly heterogeneous. Given the explosive progress in the topic and the identification of new functional types, old classifications have become outdated, raising the need for novel strategies for categorizing RBPs, RNA species, and their modes of interaction. In fact, recent efforts have been made to separate ncRNA classes [83]. Nonetheless, accommodating emerging functional types, such as short open reading frames (micropeptides), which can exist within ncRNA molecules, is still a challenge [157]. Moreover, interactions between RNA molecules and RBPs have increasingly been recognized as key mechanisms for controlling gene expression, encompassing a wide array of ncRNA molecules. These ncRNAs are crucial for various regulatory processes, including transcriptional and post-transcriptional regulation, chromatin remodeling, and DNA modifications. Notably, recent studies have highlighted the profound impact of ncRNAs on epigenetics, where they modulate gene expression by altering chromatin states and DNA methylation patterns, thus influencing cellular identity and function under both physiological and disease conditions.

On the experimental side, classical approaches to establish RNA-RBP binding events have relied on crosslinking and immunoprecipitation (CLIP) followed by sequencing or mass spectrometry to probe the transcriptome-bound (RBP-centric) or proteome-bound (RNA-centric) fractions, respectively. However, these protocols are time-consuming and scale poorly to large sets of factors, thus limiting the ability to capture the complexity of RNA-RBP regulatory networks. To circumvent some of these limitations, the field is moving toward developing novel antibody-free methods that are versatile for identifying structure- or modification-dependent interactions, highly multiplexable for profiling several factors in parallel, and extendable for reaching single-cell resolution [158–160].

In the case of computational approaches, current tools leverage sequence motifs and conservation to define structures and predict functional elements, although their performance is far from optimal. Validation experiments are still of paramount importance for determining the functional relevance of newly discovered structures and interactions. Furthermore, establishing sophisticated methods that can accurately identify RNA-RBP interactions will be essential for reconstructing complex regulatory networks. Recently, deep learning approaches have shown remarkable results for modeling protein structures [161]. However, the performance of AI-based approaches is still limited when comprehensive reference datasets, such as RNA structure and RNA-protein interaction data, are lacking [162, 163]. These approaches may also enable further stratification of elements into functional groups. As we continue to unveil RNA-RBP regulatory networks experimentally with novel methods, enough reference quality data will be generated to train the next generation of prediction tools. Here, large datasets covering multiple layers of biological information combined with integrative strategies will prove instrumental in refining the reconstructed networks and their dynamics across different conditions.

Owing to their specificity for certain cell types or disease states, the ability to robustly define regulatory networks could pave the way for novel diagnostic and therapeutic strategies. Ultimately, deciphering the complexities of RNA-RBP interactions will shed light on fundamental cellular processes while offering unique insight for clinical intervention in cancer and other diseases.

## Data Availability

No datasets were generated or analysed during the current study.

## Abbreviations

AGO: Argonaute
CBC: Cap-binding complex
circRNA: Circular RNA
ENCODE: Encyclopedia of DNA Elements
eRNA: Enhancer RNA
HCC: Hepatocellular carcinoma
lncRNA: Long noncoding RNA
m^7^G-cap: 7-methylguanosine cap
mRNA: Messenger RNA
miRNA: MicroRNA
m^6^A: N^6^-methyladenosine
Nm: 2’-O-methylation
ncRNA: Noncoding RNA
NPCs: Nuclear pore complexes
P-bodies: processing bodies
piRNA: PIWI-interacting RNA
PABP: Poly(A) binding protein
PAP: Poly(A) polymerase
poly(A): Polyadenylation
PRC2: Polycomb repressive complex 2
Pre-RNA: Precursor RNA
P-TEFb: Positive transcription elongation factor b
Ψ: Pseudouridylation
RNP: Ribonucleoprotein
rRNA: Ribosomal RNA
Pol: RNA polymerase
RBD: RNA-binding domain
RBP: RNA-binding protein
snRNA: Small nuclear RNA
snoRNA: Small nucleolar RNA
tRNA: Transfer RNA
tRF: TRNA-derived fragment
UTR: Untranslated region

## References

[1] Alberts B, Wilson JH, Hunt T. Molecular biology of the cell. 5th ed. New York: Garland Science; 2008. xxxiii, 1601, 90 p. p.

[2] Skalska L, Beltran-Nebot M, Ule J, Jenner RG. Regulatory feedback from nascent RNA to chromatin and transcription. Nat Rev Mol Cell Biol. 2017;18(5):331–7. 28270684 10.1038/nrm.2017.12

[3] Van Nostrand EL, Freese P, Pratt GA, Wang X, Wei X, Xiao R, et al. A large-scale binding and functional map of human RNA-binding proteins. Nature. 2020;583(7818):711–9. 32728246 10.1038/s41586-020-2077-3 PMC7410833

[4] Cooper TA, Wan L, Dreyfuss G. RNA and disease. Cell. 2009;136(4):777–93. 19239895 10.1016/j.cell.2009.02.011 PMC2866189

[5] Gebauer F, Schwarzl T, Valcarcel J, Hentze MW. RNA-binding proteins in human genetic disease. Nat Rev Genet. 2021;22(3):185–98. 33235359 10.1038/s41576-020-00302-y

[6] Consortium EP. An integrated encyclopedia of DNA elements in the human genome. Nature. 2012;489(7414):57–74. 22955616 10.1038/nature11247 PMC3439153

[7] Frith MC, Pheasant M, Mattick JS. The amazing complexity of the human transcriptome. Eur J Hum Genet. 2005;13(8):894–7. 15970949 10.1038/sj.ejhg.5201459

[8] International Human Genome Sequencing C. Finishing the euchromatic sequence of the human genome. Nature. 2004;431(7011):931–45. 15496913 10.1038/nature03001

[9] Nurk S, Koren S, Rhie A, Rautiainen M, Bzikadze AV, Mikheenko A, et al. The complete sequence of a human genome. Science. 2022;376(6588):44–53. 35357919 10.1126/science.abj6987 PMC9186530

[10] Christmas MJ, Kaplow IM, Genereux DP, Dong MX, Hughes GM, Li X, et al. Evolutionary constraint and innovation across hundreds of placental mammals. Science. 2023;380(6643):eabn3943. 37104599 10.1126/science.abn3943 PMC10250106

[11] Galeota-Sprung B, Sniegowski P, Ewens W. Mutational load and the functional fraction of the Human Genome. Genome Biol Evol. 2020;12(4):273–81. 32108234 10.1093/gbe/evaa040 PMC7151545

[12] Meader S, Ponting CP, Lunter G. Massive turnover of functional sequence in human and other mammalian genomes. Genome Res. 2010;20(10):1335–43. 20693480 10.1101/gr.108795.110 PMC2945182

[13] Pheasant M, Mattick JS. Raising the estimate of functional human sequences. Genome Res. 2007;17(9):1245–53. 17690206 10.1101/gr.6406307

[14] Leypold NA, Speicher MR. Evolutionary conservation in noncoding genomic regions. Trends Genet. 2021;37(10):903–18. 34238591 10.1016/j.tig.2021.06.007

[15] Pang KC, Frith MC, Mattick JS. Rapid evolution of noncoding RNAs: lack of conservation does not mean lack of function. Trends Genet. 2006;22(1):1–5. 16290135 10.1016/j.tig.2005.10.003

[16] Kutter C, Brown GD, Goncalves A, Wilson MD, Watt S, Brazma A, et al. Pol III binding in six mammals shows conservation among amino acid isotypes despite divergence among tRNA genes. Nat Genet. 2011;43(10):948–55. 21873999 10.1038/ng.906 PMC3184141

[17] Kutter C, Watt S, Stefflova K, Wilson MD, Goncalves A, Ponting CP, et al. Rapid turnover of long noncoding RNAs and the evolution of gene expression. PLoS Genet. 2012;8(7):e1002841. 22844254 10.1371/journal.pgen.1002841 PMC3406015

[18] Calin GA, Dumitru CD, Shimizu M, Bichi R, Zupo S, Noch E, et al. Frequent deletions and down-regulation of micro- RNA genes miR15 and miR16 at 13q14 in chronic lymphocytic leukemia. Proc Natl Acad Sci U S A. 2002;99(24):15524–9. 12434020 10.1073/pnas.242606799 PMC137750

[19] Huppertz I, Perez-Perri JI, Mantas P, Sekaran T, Schwarzl T, Russo F, et al. Riboregulation of Enolase 1 activity controls glycolysis and embryonic stem cell differentiation. Mol Cell. 2022;82(14):2666–80. e11. 35709751 10.1016/j.molcel.2022.05.019

[20] Kim J, Piao HL, Kim BJ, Yao F, Han Z, Wang Y, et al. Long noncoding RNA MALAT1 suppresses breast cancer metastasis. Nat Genet. 2018;50(12):1705–15. 30349115 10.1038/s41588-018-0252-3 PMC6265076

[21] Rom A, Melamed L, Gil N, Goldrich MJ, Kadir R, Golan M, et al. Regulation of CHD2 expression by the Chaserr long noncoding RNA gene is essential for viability. Nat Commun. 2019;10(1):5092. 31704914 10.1038/s41467-019-13075-8 PMC6841665

[22] Sommerauer C, Kutter C. Noncoding RNAs and RNA-binding proteins: emerging governors of liver physiology and metabolic diseases. Am J Physiol Cell Physiol. 2022;323(4):C1003–17. 35968891 10.1152/ajpcell.00232.2022

[23] Sondergaard JN, Sommerauer C, Atanasoai I, Hinte LC, Geng K, Guiducci G, et al. CCT3-LINC00326 axis regulates hepatocarcinogenic lipid metabolism. Gut. 2022;71(10):2081–92. 35022268 10.1136/gutjnl-2021-325109 PMC9484377

[24] Aviran S, Incarnato D. Computational approaches for RNA structure ensemble deconvolution from structure probing data. J Mol Biol. 2022;434(18):167635. 35595163 10.1016/j.jmb.2022.167635

[25] Morandi E, van Hemert MJ, Incarnato D. SHAPE-guided RNA structure homology search and motif discovery. Nat Commun. 2022;13(1):1722. 35361788 10.1038/s41467-022-29398-y PMC8971488

[26] Spitale RC, Incarnato D. Probing the dynamic RNA structurome and its functions. Nat Rev Genet. 2023;24(3):178–96. 36348050 10.1038/s41576-022-00546-w PMC9644009

[27] Gao W, Yang A, Rivas E. Thirteen dubious ways to detect conserved structural RNAs. IUBMB Life. 2023;75(6):471–92. 36495545 10.1002/iub.2694 PMC11234323

[28] Hentze MW, Castello A, Schwarzl T, Preiss T. A brave new world of RNA-binding proteins. Nat Rev Mol Cell Biol. 2018;19(5):327–41. 29339797 10.1038/nrm.2017.130

[29] Lunde BM, Moore C, Varani G. RNA-binding proteins: modular design for efficient function. Nat Rev Mol Cell Biol. 2007;8(6):479–90. 17473849 10.1038/nrm2178 PMC5507177

[30] Castello A, Fischer B, Eichelbaum K, Horos R, Beckmann BM, Strein C, et al. Insights into RNA biology from an atlas of mammalian mRNA-binding proteins. Cell. 2012;149(6):1393–406. 22658674 10.1016/j.cell.2012.04.031

[31] Gerstberger S, Hafner M, Tuschl T. A census of human RNA-binding proteins. Nat Rev Genet. 2014;15(12):829–45. 25365966 10.1038/nrg3813 PMC11148870

[32] Queiroz RML, Smith T, Villanueva E, Marti-Solano M, Monti M, Pizzinga M, et al. Comprehensive identification of RNA-protein interactions in any organism using orthogonal organic phase separation (OOPS). Nat Biotechnol. 2019;37(2):169–78. 30607034 10.1038/s41587-018-0001-2 PMC6591131

[33] Trendel J, Schwarzl T, Horos R, Prakash A, Bateman A, Hentze MW, et al. The human RNA-Binding proteome and its dynamics during translational arrest. Cell. 2019;176(1–2):391–403. e19. 30528433 10.1016/j.cell.2018.11.004

[34] Zhao W, Zhang S, Zhu Y, Xi X, Bao P, Ma Z, et al. POSTAR3: an updated platform for exploring post-transcriptional regulation coordinated by RNA-binding proteins. Nucleic Acids Res. 2022;50(D1):D287–94. 34403477 10.1093/nar/gkab702 PMC8728292

[35] Liao JY, Yang B, Zhang YC, Wang XJ, Ye Y, Peng JW, et al. EuRBPDB: a comprehensive resource for annotation, functional and oncological investigation of eukaryotic RNA binding proteins (RBPs). Nucleic Acids Res. 2020;48(D1):D307–13. 31598693 10.1093/nar/gkz823 PMC6943034

[36] Li JH, Liu S, Zhou H, Qu LH, Yang JH. starBase v2.0: decoding miRNA-ceRNA, miRNA-ncRNA and protein-RNA interaction networks from large-scale CLIP-Seq data. Nucleic Acids Res. 2014;42(Database issue):D92–7. 24297251 10.1093/nar/gkt1248 PMC3964941

[37] Kang J, Tang Q, He J, Li L, Yang N, Yu S, et al. RNAInter v4.0: RNA interactome repository with redefined confidence scoring system and improved accessibility. Nucleic Acids Res. 2022;50(D1):D326–32. 34718726 10.1093/nar/gkab997 PMC8728132

[38] He C, Sidoli S, Warneford-Thomson R, Tatomer DC, Wilusz JE, Garcia BA, et al. High-resolution mapping of RNA-Binding regions in the Nuclear Proteome of embryonic stem cells. Mol Cell. 2016;64(2):416–30. 27768875 10.1016/j.molcel.2016.09.034 PMC5222606

[39] Cook KB, Kazan H, Zuberi K, Morris Q, Hughes TR. RBPDB: a database of RNA-binding specificities. Nucleic Acids Res. 2011;39(Database issue):D301–8. 21036867 10.1093/nar/gkq1069 PMC3013675

[40] Caudron-Herger M, Jansen RE, Wassmer E, Diederichs S. RBP2GO: a comprehensive pan-species database on RNA-binding proteins, their interactions and functions. Nucleic Acids Res. 2021;49(D1):D425–36. 33196814 10.1093/nar/gkaa1040 PMC7778890

[41] Bheemireddy S, Sandhya S, Srinivasan N, Sowdhamini R. Computational tools to study RNA-protein complexes. Front Mol Biosci. 2022;9:954926. 36275618 10.3389/fmolb.2022.954926 PMC9585174

[42] Moore KS, t Hoen PAC. Computational approaches for the analysis of RNA-protein interactions: a primer for biologists. J Biol Chem. 2019;294(1):1–9. 30455357 10.1074/jbc.REV118.004842 PMC6322881

[43] Gerstberger S, Hafner M, Ascano M, Tuschl T. Evolutionary conservation and expression of human RNA-binding proteins and their role in human genetic disease. Adv Exp Med Biol. 2014;825:1–55. 25201102 10.1007/978-1-4939-1221-6_1 PMC4180674

[44] Lukong KE, Chang KW, Khandjian EW, Richard S. RNA-binding proteins in human genetic disease. Trends Genet. 2008;24(8):416–25. 18597886 10.1016/j.tig.2008.05.004

[45] Cech TR, Steitz JA. The noncoding RNA revolution-trashing old rules to forge new ones. Cell. 2014;157(1):77–94. 24679528 10.1016/j.cell.2014.03.008

[46] Huttenhofer A, Schattner P, Polacek N. Non-coding RNAs: hope or hype? Trends Genet. 2005;21(5):289–97. 15851066 10.1016/j.tig.2005.03.007

[47] Ma L, Bajic VB, Zhang Z. On the classification of long non-coding RNAs. RNA Biol. 2013;10(6):925–33. 23696037 10.4161/rna.24604 PMC4111732

[48] Zhang P, Wu W, Chen Q, Chen M. Non-coding RNAs and their Integrated Networks. J Integr Bioinform. 2019;16(3). 10.1515/jib-2019-0027 PMC6798851 31301674

[49] Barba-Aliaga M, Alepuz P, Perez-Ortin JE. Eukaryotic RNA polymerases: the many ways to transcribe a gene. Front Mol Biosci. 2021;8:663209. 33968992 10.3389/fmolb.2021.663209 PMC8097091

[50] Girbig M, Misiaszek AD, Muller CW. Structural insights into nuclear transcription by eukaryotic DNA-dependent RNA polymerases. Nat Rev Mol Cell Biol. 2022;23(9):603–22. 35505252 10.1038/s41580-022-00476-9

[51] Wang KC, Yang YW, Liu B, Sanyal A, Corces-Zimmerman R, Chen Y, et al. A long noncoding RNA maintains active chromatin to coordinate homeotic gene expression. Nature. 2011;472(7341):120–4. 21423168 10.1038/nature09819 PMC3670758

[52] Brockdorff N. X-chromosome inactivation: closing in on proteins that bind xist RNA. Trends Genet. 2002;18(7):352–8. 12127775 10.1016/s0168-9525(02)02717-8

[53] Loda A, Collombet S, Heard E. Gene regulation in time and space during X-chromosome inactivation. Nat Rev Mol Cell Biol. 2022;23(4):231–49. 35013589 10.1038/s41580-021-00438-7

[54] Long Y, Hwang T, Gooding AR, Goodrich KJ, Rinn JL, Cech TR. RNA is essential for PRC2 chromatin occupancy and function in human pluripotent stem cells. Nat Genet. 2020;52(9):931–8. 32632336 10.1038/s41588-020-0662-x PMC10353856

[55] Nielsen M, Ulitksy I. The links are still missing: revisiting the role of RNA as a guide for chromatin-associated proteins. Mol Cell. 2024;84(7):1178–9. 38579673 10.1016/j.molcel.2024.03.005

[56] Palazzo AF, Lee ES. Non-coding RNA: what is functional and what is junk? Front Genet. 2015;6:2. 25674102 10.3389/fgene.2015.00002 PMC4306305

[57] Lee TI, Young RA. Transcription of eukaryotic protein-coding genes. Annu Rev Genet. 2000;34:77–137. 11092823 10.1146/annurev.genet.34.1.77

[58] Hinnebusch AG, Lorsch JR. The mechanism of eukaryotic translation initiation: new insights and challenges. Cold Spring Harb Perspect Biol. 2012;4(10). 10.1101/cshperspect.a011544 PMC3475172 22815232

[59] Lopes I, Altab G, Raina P, de Magalhaes JP. Gene size matters: an analysis of gene length in the Human Genome. Front Genet. 2021;12:559998. 33643374 10.3389/fgene.2021.559998 PMC7905317

[60] Khatter H, Myasnikov AG, Natchiar SK, Klaholz BP. Structure of the human 80S ribosome. Nature. 2015;520(7549):640–5. 25901680 10.1038/nature14427

[61] Fedoriw AM, Starmer J, Yee D, Magnuson T. Nucleolar association and transcriptional inhibition through 5S rDNA in mammals. PLoS Genet. 2012;8(1):e1002468. 22275877 10.1371/journal.pgen.1002468 PMC3261910

[62] Nerurkar P, Altvater M, Gerhardy S, Schutz S, Fischer U, Weirich C, et al. Eukaryotic Ribosome Assembly and Nuclear Export. Int Rev Cell Mol Biol. 2015;319:107–40. 26404467 10.1016/bs.ircmb.2015.07.002

[63] Paule MR, White RJ. Survey and summary: transcription by RNA polymerases I and III. Nucleic Acids Res. 2000;28(6):1283–98. 10684922 10.1093/nar/28.6.1283 PMC111039

[64] Henras AK, Plisson-Chastang C, O’Donohue MF, Chakraborty A, Gleizes PE. An overview of pre-ribosomal RNA processing in eukaryotes. Wiley Interdiscip Rev RNA. 2015;6(2):225–42. 25346433 10.1002/wrna.1269 PMC4361047

[65] Dever TE, Dinman JD, Green R. Translation elongation and recoding in eukaryotes. Cold Spring Harb Perspect Biol. 2018;10(8). 10.1101/cshperspect.a032649 PMC6071482 29610120

[66] Chan PP, Lowe TM. GtRNAdb 2.0: an expanded database of transfer RNA genes identified in complete and draft genomes. Nucleic Acids Res. 2016;44(D1):D184–9. 26673694 10.1093/nar/gkv1309 PMC4702915

[67] Dieci G, Fiorino G, Castelnuovo M, Teichmann M, Pagano A. The expanding RNA polymerase III transcriptome. Trends Genet. 2007;23(12):614–22. 17977614 10.1016/j.tig.2007.09.001

[68] Goodenbour JM, Pan T. Diversity of tRNA genes in eukaryotes. Nucleic Acids Res. 2006;34(21):6137–46. 17088292 10.1093/nar/gkl725 PMC1693877

[69] Goodarzi H, Liu X, Nguyen HC, Zhang S, Fish L, Tavazoie SF. Endogenous tRNA-Derived fragments suppress breast Cancer progression via YBX1 displacement. Cell. 2015;161(4):790–802. 25957686 10.1016/j.cell.2015.02.053 PMC4457382

[70] Lee YS, Shibata Y, Malhotra A, Dutta A. A novel class of small RNAs: tRNA-derived RNA fragments (tRFs). Genes Dev. 2009;23(22):2639–49. 19933153 10.1101/gad.1837609 PMC2779758

[71] Schorn AJ, Gutbrod MJ, LeBlanc C, Martienssen R. LTR-Retrotransposon control by tRNA-Derived small RNAs. Cell. 2017;170(1):61–e7111. 28666125 10.1016/j.cell.2017.06.013 PMC5551035

[72] Matera AG, Wang Z. A day in the life of the spliceosome. Nat Rev Mol Cell Biol. 2014;15(2):108–21. 24452469 10.1038/nrm3742 PMC4060434

[73] Valadkhan S, Gunawardane LS. Role of small nuclear RNAs in eukaryotic gene expression. Essays Biochem. 2013;54:79–90. 23829528 10.1042/bse0540079 PMC11246792

[74] Bratkovic T, Bozic J, Rogelj B. Functional diversity of small nucleolar RNAs. Nucleic Acids Res. 2020;48(4):1627–51. 31828325 10.1093/nar/gkz1140 PMC7038934

[75] Bergeron D, Paraqindes H, Fafard-Couture E, Deschamps-Francoeur G, Faucher-Giguere L, Bouchard-Bourelle P, et al. snoDB 2.0: an enhanced interactive database, specializing in human snoRNAs. Nucleic Acids Res. 2023;51(D1):D291–6. 36165892 10.1093/nar/gkac835 PMC9825428

[76] Kufel J, Grzechnik P. Small nucleolar RNAs tell a different tale. Trends Genet. 2019;35(2):104–17. 30563726 10.1016/j.tig.2018.11.005

[77] Lee RC, Feinbaum RL, Ambros V. The C. Elegans heterochronic gene lin-4 encodes small RNAs with antisense complementarity to lin-14. Cell. 1993;75(5):843–54. 8252621 10.1016/0092-8674(93)90529-y

[78] Bartel DP. MicroRNAs: genomics, biogenesis, mechanism, and function. Cell. 2004;116(2):281–97. 14744438 10.1016/s0092-8674(04)00045-5

[79] Ha M, Kim VN. Regulation of microRNA biogenesis. Nat Rev Mol Cell Biol. 2014;15(8):509–24. 25027649 10.1038/nrm3838

[80] Babiarz JE, Ruby JG, Wang Y, Bartel DP, Blelloch R. Mouse ES cells express endogenous shRNAs, siRNAs, and other Microprocessor-independent, dicer-dependent small RNAs. Genes Dev. 2008;22(20):2773–85. 18923076 10.1101/gad.1705308 PMC2569885

[81] Ernst C, Odom DT, Kutter C. The emergence of piRNAs against transposon invasion to preserve mammalian genome integrity. Nat Commun. 2017;8(1):1411. 29127279 10.1038/s41467-017-01049-7 PMC5681665

[82] Frankish A, Carbonell-Sala S, Diekhans M, Jungreis I, Loveland JE, Mudge JM, et al. GENCODE: reference annotation for the human and mouse genomes in 2023. Nucleic Acids Res. 2023;51(D1):D942–9. 36420896 10.1093/nar/gkac1071 PMC9825462

[83] Mattick JS, Amaral PP, Carninci P, Carpenter S, Chang HY, Chen LL, et al. Long non-coding RNAs: definitions, functions, challenges and recommendations. Nat Rev Mol Cell Biol. 2023;24(6):430–47. 36596869 10.1038/s41580-022-00566-8 PMC10213152

[84] Statello L, Guo CJ, Chen LL, Huarte M. Gene regulation by long non-coding RNAs and its biological functions. Nat Rev Mol Cell Biol. 2021;22(2):96–118. 33353982 10.1038/s41580-020-00315-9 PMC7754182

[85] Tilgner H, Knowles DG, Johnson R, Davis CA, Chakrabortty S, Djebali S, et al. Deep sequencing of subcellular RNA fractions shows splicing to be predominantly co-transcriptional in the human genome but inefficient for lncRNAs. Genome Res. 2012;22(9):1616–25. 22955974 10.1101/gr.134445.111 PMC3431479

[86] Memczak S, Jens M, Elefsinioti A, Torti F, Krueger J, Rybak A, et al. Circular RNAs are a large class of animal RNAs with regulatory potency. Nature. 2013;495(7441):333–8. 23446348 10.1038/nature11928

[87] Patop IL, Wust S, Kadener S. Past, present, and future of circRNAs. EMBO J. 2019;38(16):e100836. 31343080 10.15252/embj.2018100836 PMC6694216

[88] De Santa F, Barozzi I, Mietton F, Ghisletti S, Polletti S, Tusi BK, et al. A large fraction of extragenic RNA pol II transcription sites overlap enhancers. PLoS Biol. 2010;8(5):e1000384. 20485488 10.1371/journal.pbio.1000384 PMC2867938

[89] Sartorelli V, Lauberth SM. Enhancer RNAs are an important regulatory layer of the epigenome. Nat Struct Mol Biol. 2020;27(6):521–8. 32514177 10.1038/s41594-020-0446-0 PMC7343394

[90] Levengood JD, Potoyan D, Penumutchu S, Kumar A, Zhou Q, Wang Y, et al. Thermodynamic coupling of the tandem RRM domains of hnRNP A1 underlie its pleiotropic RNA binding functions. Sci Adv. 2024;10(28):eadk6580. 38985864 10.1126/sciadv.adk6580 PMC11235170

[91] McCracken S, Fong N, Rosonina E, Yankulov K, Brothers G, Siderovski D, et al. 5’-Capping enzymes are targeted to pre-mRNA by binding to the phosphorylated carboxy-terminal domain of RNA polymerase II. Genes Dev. 1997;11(24):3306–18. 9407024 10.1101/gad.11.24.3306 PMC316822

[92] Ramanathan A, Robb GB, Chan SH. mRNA capping: biological functions and applications. Nucleic Acids Res. 2016;44(16):7511–26. 27317694 10.1093/nar/gkw551 PMC5027499

[93] Nguyen VT, Kiss T, Michels AA, Bensaude O. 7SK small nuclear RNA binds to and inhibits the activity of CDK9/cyclin T complexes. Nature. 2001;414(6861):322–5. 11713533 10.1038/35104581

[94] Peterlin BM, Brogie JE, Price DH. 7SK snRNA: a noncoding RNA that plays a major role in regulating eukaryotic transcription. Wiley Interdiscip Rev RNA. 2012;3(1):92–103. 21853533 10.1002/wrna.106 PMC3223291

[95] Herzel L, Ottoz DSM, Alpert T, Neugebauer KM. Splicing and transcription touch base: co-transcriptional spliceosome assembly and function. Nat Rev Mol Cell Biol. 2017;18(10):637–50. 28792005 10.1038/nrm.2017.63 PMC5928008

[96] Oesterreich FC, Herzel L, Straube K, Hujer K, Howard J, Neugebauer KM. Splicing of nascent RNA coincides with Intron exit from RNA polymerase II. Cell. 2016;165(2):372–81. 27020755 10.1016/j.cell.2016.02.045 PMC4826323

[97] Wang Z, Burge CB. Splicing regulation: from a parts list of regulatory elements to an integrated splicing code. RNA. 2008;14(5):802–13. 18369186 10.1261/rna.876308 PMC2327353

[98] Barbosa-Morais NL, Irimia M, Pan Q, Xiong HY, Gueroussov S, Lee LJ, et al. The evolutionary landscape of alternative splicing in vertebrate species. Science. 2012;338(6114):1587–93. 23258890 10.1126/science.1230612

[99] Lewis CJ, Pan T, Kalsotra A. RNA modifications and structures cooperate to guide RNA-protein interactions. Nat Rev Mol Cell Biol. 2017;18(3):202–10. 28144031 10.1038/nrm.2016.163 PMC5542016

[100] Malla S, Prasad Bhattarai D, Groza P, Melguizo-Sanchis D, Atanasoai I, Martinez-Gamero C, et al. ZFP207 sustains pluripotency by coordinating OCT4 stability, alternative splicing and RNA export. EMBO Rep. 2022;23(3):e53191. 35037361 10.15252/embr.202153191 PMC8892232

[101] Zhou KI, Shi H, Lyu R, Wylder AC, Matuszek Z, Pan JN, et al. Regulation of co-transcriptional Pre-mRNA splicing by m(6)a through the low-complexity protein hnRNPG. Mol Cell. 2019;76(1):70–e819. 31445886 10.1016/j.molcel.2019.07.005 PMC6778029

[102] Gruber AJ, Zavolan M. Alternative cleavage and polyadenylation in health and disease. Nat Rev Genet. 2019;20(10):599–614. 31267064 10.1038/s41576-019-0145-z

[103] Hill CH, Boreikaite V, Kumar A, Casanal A, Kubik P, Degliesposti G, et al. Activation of the endonuclease that defines mRNA 3’ ends requires incorporation into an 8-Subunit core cleavage and polyadenylation factor complex. Mol Cell. 2019;73(6):1217–e3111. 30737185 10.1016/j.molcel.2018.12.023 PMC6436931

[104] Tian B, Manley JL. Alternative polyadenylation of mRNA precursors. Nat Rev Mol Cell Biol. 2017;18(1):18–30. 27677860 10.1038/nrm.2016.116 PMC5483950

[105] Kuhn U, Gundel M, Knoth A, Kerwitz Y, Rudel S, Wahle E. Poly(A) tail length is controlled by the nuclear poly(A)-binding protein regulating the interaction between poly(A) polymerase and the cleavage and polyadenylation specificity factor. J Biol Chem. 2009;284(34):22803–14. 19509282 10.1074/jbc.M109.018226 PMC2755688

[106] Aspden JL, Wallace EWJ, Whiffin N. Not all exons are protein coding: addressing a common misconception. Cell Genom. 2023;3(4):100296. 37082142 10.1016/j.xgen.2023.100296 PMC10112331

[107] Hinnebusch AG, Ivanov IP, Sonenberg N. Translational control by 5’-untranslated regions of eukaryotic mRNAs. Science. 2016;352(6292):1413–6. 27313038 10.1126/science.aad9868 PMC7422601

[108] Weber R, Ghoshdastider U, Spies D, Dure C, Valdivia-Francia F, Forny M, et al. Monitoring the 5’UTR landscape reveals isoform switches to drive translational efficiencies in cancer. Oncogene. 2023;42(9):638–50. 36550360 10.1038/s41388-022-02578-2 PMC9957725

[109] Berkovits BD, Mayr C. Alternative 3’ UTRs act as scaffolds to regulate membrane protein localization. Nature. 2015;522(7556):363–7. 25896326 10.1038/nature14321 PMC4697748

[110] Xiang K, Ly J, Bartel DP. Control of poly(A)-tail length and translation in vertebrate oocytes and early embryos. Dev Cell. 2024;59(8):1058–e7411. 38460509 10.1016/j.devcel.2024.02.007

[111] Guo CJ, Ma XK, Xing YH, Zheng CC, Xu YF, Shan L, et al. Distinct Processing of lncRNAs contributes to non-conserved functions in stem cells. Cell. 2020;181(3):621–36. e22. 32259487 10.1016/j.cell.2020.03.006

[112] Grelet S, Link LA, Howley B, Obellianne C, Palanisamy V, Gangaraju VK, et al. A regulated PNUTS mRNA to lncRNA splice switch mediates EMT and tumour progression. Nat Cell Biol. 2017;19(9):1105–15. 28825698 10.1038/ncb3595 PMC5578890

[113] Wickramasinghe VO, Laskey RA. Control of mammalian gene expression by selective mRNA export. Nat Rev Mol Cell Biol. 2015;16(7):431–42. 26081607 10.1038/nrm4010

[114] Preiss T, Hentze MW. Dual function of the messenger RNA cap structure in poly(A)-tail-promoted translation in yeast. Nature. 1998;392(6675):516–20. 9548259 10.1038/33192

[115] Quax TE, Claassens NJ, Soll D, van der Oost J. Codon Bias as a Means to Fine-Tune Gene expression. Mol Cell. 2015;59(2):149–61. 26186290 10.1016/j.molcel.2015.05.035 PMC4794256

[116] Tuller T, Carmi A, Vestsigian K, Navon S, Dorfan Y, Zaborske J, et al. An evolutionarily conserved mechanism for controlling the efficiency of protein translation. Cell. 2010;141(2):344–54. 20403328 10.1016/j.cell.2010.03.031

[117] Rudolph KL, Schmitt BM, Villar D, White RJ, Marioni JC, Kutter C, et al. Codon-Driven Translational Efficiency is stable across diverse mammalian cell States. PLoS Genet. 2016;12(5):e1006024. 27166679 10.1371/journal.pgen.1006024 PMC4864286

[118] Garneau NL, Wilusz J, Wilusz CJ. The highways and byways of mRNA decay. Nat Rev Mol Cell Biol. 2007;8(2):113–26. 17245413 10.1038/nrm2104

[119] Behm-Ansmant I, Rehwinkel J, Doerks T, Stark A, Bork P, Izaurralde E. mRNA degradation by miRNAs and GW182 requires both CCR4:NOT deadenylase and DCP1:DCP2 decapping complexes. Genes Dev. 2006;20(14):1885–98. 16815998 10.1101/gad.1424106 PMC1522082

[120] Eichhorn SW, Guo H, McGeary SE, Rodriguez-Mias RA, Shin C, Baek D, et al. mRNA destabilization is the dominant effect of mammalian microRNAs by the time substantial repression ensues. Mol Cell. 2014;56(1):104–15. 25263593 10.1016/j.molcel.2014.08.028 PMC4292926

[121] Zeng Y, Yi R, Cullen BR. MicroRNAs and small interfering RNAs can inhibit mRNA expression by similar mechanisms. Proc Natl Acad Sci U S A. 2003;100(17):9779–84. 12902540 10.1073/pnas.1630797100 PMC187842

[122] Wang X, Lu Z, Gomez A, Hon GC, Yue Y, Han D, et al. N6-methyladenosine-dependent regulation of messenger RNA stability. Nature. 2014;505(7481):117–20. 24284625 10.1038/nature12730 PMC3877715

[123] Katz ZB, Wells AL, Park HY, Wu B, Shenoy SM, Singer RH. beta-actin mRNA compartmentalization enhances focal adhesion stability and directs cell migration. Genes Dev. 2012;26(17):1885–90. 22948660 10.1101/gad.190413.112 PMC3435492

[124] Buchan JR. mRNP granules. Assembly, function, and connections with disease. RNA Biol. 2014;11(8):1019–30. 25531407 10.4161/15476286.2014.972208 PMC4615263

[125] Ripin N, Parker R. Formation, function, and pathology of RNP granules. Cell. 2023;186(22):4737–56. 37890457 10.1016/j.cell.2023.09.006 PMC10617657

[126] Kechavarzi B, Janga SC. Dissecting the expression landscape of RNA-binding proteins in human cancers. Genome Biol. 2014;15(1):R14. 24410894 10.1186/gb-2014-15-1-r14 PMC4053825

[127] Wang ZL, Li B, Luo YX, Lin Q, Liu SR, Zhang XQ, et al. Comprehensive genomic characterization of RNA-Binding proteins across human cancers. Cell Rep. 2018;22(1):286–98. 29298429 10.1016/j.celrep.2017.12.035

[128] Wang M, Huang S, Chen Z, Han Z, Li K, Chen C, et al. Development and validation of an RNA binding protein-associated prognostic model for hepatocellular carcinoma. BMC Cancer. 2020;20(1):1136. 33228611 10.1186/s12885-020-07625-3 PMC7684760

[129] Mizutani R, Imamachi N, Suzuki Y, Yoshida H, Tochigi N, Oonishi T, et al. Oncofetal protein IGF2BP3 facilitates the activity of proto-oncogene protein eIF4E through the destabilization of EIF4E-BP2 mRNA. Oncogene. 2016;35(27):3495–502. 26522719 10.1038/onc.2015.410

[130] Sysoev VO, Fischer B, Frese CK, Gupta I, Krijgsveld J, Hentze MW, et al. Global changes of the RNA-bound proteome during the maternal-to-zygotic transition in Drosophila. Nat Commun. 2016;7:12128. 27378189 10.1038/ncomms12128 PMC4935972

[131] Lee LJ, Papadopoli D, Jewer M, Del Rincon S, Topisirovic I, Lawrence MG, et al. Cancer plasticity: the role of mRNA translation. Trends Cancer. 2021;7(2):134–45. 33067172 10.1016/j.trecan.2020.09.005 PMC8023421

[132] Gao W, Gallardo-Dodd CJ, Kutter C. Cell type-specific analysis by single-cell profiling identifies a stable mammalian tRNA-mRNA interface and increased translation efficiency in neurons. Genome Res. 2022;32(1):97–110. 34857654 10.1101/gr.275944.121 PMC8744671

[133] Schmitt BM, Rudolph KL, Karagianni P, Fonseca NA, White RJ, Talianidis I, et al. High-resolution mapping of transcriptional dynamics across tissue development reveals a stable mRNA-tRNA interface. Genome Res. 2014;24(11):1797–807. 25122613 10.1101/gr.176784.114 PMC4216921

[134] Presnyak V, Alhusaini N, Chen YH, Martin S, Morris N, Kline N, et al. Codon optimality is a major determinant of mRNA stability. Cell. 2015;160(6):1111–24. 25768907 10.1016/j.cell.2015.02.029 PMC4359748

[135] Goodarzi H, Nguyen HCB, Zhang S, Dill BD, Molina H, Tavazoie SF. Modulated expression of specific tRNAs drives Gene expression and Cancer progression. Cell. 2016;165(6):1416–27. 27259150 10.1016/j.cell.2016.05.046 PMC4915377

[136] Zhang Z, Ye Y, Gong J, Ruan H, Liu CJ, Xiang Y, et al. Global analysis of tRNA and translation factor expression reveals a dynamic landscape of translational regulation in human cancers. Commun Biol. 2018;1:234. 30588513 10.1038/s42003-018-0239-8 PMC6303286

[137] Uemura M, Zheng Q, Koh CM, Nelson WG, Yegnasubramanian S, De Marzo AM. Overexpression of ribosomal RNA in prostate cancer is common but not linked to rDNA promoter hypomethylation. Oncogene. 2012;31(10):1254–63. 21822302 10.1038/onc.2011.319 PMC3298623

[138] Zhou H, Wang Y, Lv Q, Zhang J, Wang Q, Gao F, et al. Overexpression of ribosomal RNA in the development of human cervical Cancer is Associated with rDNA promoter hypomethylation. PLoS ONE. 2016;11(10):e0163340. 27695092 10.1371/journal.pone.0163340 PMC5047480

[139] Jack K, Bellodi C, Landry DM, Niederer RO, Meskauskas A, Musalgaonkar S, et al. rRNA pseudouridylation defects affect ribosomal ligand binding and translational fidelity from yeast to human cells. Mol Cell. 2011;44(4):660–6. 22099312 10.1016/j.molcel.2011.09.017 PMC3222873

[140] Marcel V, Ghayad SE, Belin S, Therizols G, Morel AP, Solano-Gonzalez E, et al. p53 acts as a safeguard of translational control by regulating fibrillarin and rRNA methylation in cancer. Cancer Cell. 2013;24(3):318–30. 24029231 10.1016/j.ccr.2013.08.013 PMC7106277

[141] Zhang M, Li K, Bai J, Van Damme R, Zhang W, Alba M, et al. A snoRNA-tRNA modification network governs codon-biased cellular states. Proc Natl Acad Sci U S A. 2023;120(41):e2312126120. 37792516 10.1073/pnas.2312126120 PMC10576143

[142] Kim HK, Fuchs G, Wang S, Wei W, Zhang Y, Park H, et al. A transfer-RNA-derived small RNA regulates ribosome biogenesis. Nature. 2017;552(7683):57–62. 29186115 10.1038/nature25005 PMC6066594

[143] Meng F, Henson R, Wehbe-Janek H, Ghoshal K, Jacob ST, Patel T. MicroRNA-21 regulates expression of the PTEN tumor suppressor gene in human hepatocellular cancer. Gastroenterology. 2007;133(2):647–58. 17681183 10.1053/j.gastro.2007.05.022 PMC4285346

[144] Bautista-Sanchez D, Arriaga-Canon C, Pedroza-Torres A, De La Rosa-Velazquez IA, Gonzalez-Barrios R, Contreras-Espinosa L, et al. The promising role of miR-21 as a Cancer Biomarker and its importance in RNA-Based therapeutics. Mol Ther Nucleic Acids. 2020;20:409–20. 32244168 10.1016/j.omtn.2020.03.003 PMC7118281

[145] Sampson VB, Rong NH, Han J, Yang Q, Aris V, Soteropoulos P, et al. MicroRNA let-7a down-regulates MYC and reverts MYC-induced growth in Burkitt lymphoma cells. Cancer Res. 2007;67(20):9762–70. 17942906 10.1158/0008-5472.CAN-07-2462

[146] Li Z, Li Y, Li Y, Ren K, Li X, Han X et al. Long non-coding RNA H19 promotes the proliferation and invasion of breast cancer through upregulating DNMT1 expression by sponging miR-152. J Biochem Mol Toxicol. 2017;31(9). 10.1002/jbt.21933 28544374

[147] Liu L, Yang J, Zhu X, Li D, Lv Z, Zhang X. Long noncoding RNA H19 competitively binds mir-17-5p to regulate YES1 expression in thyroid cancer. FEBS J. 2016;283(12):2326–39. 27093644 10.1111/febs.13741

[148] Prensner JR, Chen W, Han S, Iyer MK, Cao Q, Kothari V, et al. The long non-coding RNA PCAT-1 promotes prostate cancer cell proliferation through cMyc. Neoplasia. 2014;16(11):900–8. 25425964 10.1016/j.neo.2014.09.001 PMC4240923

[149] Lee S, Kopp F, Chang TC, Sataluri A, Chen B, Sivakumar S, et al. Noncoding RNA NORAD regulates genomic Stability by Sequestering PUMILIO proteins. Cell. 2016;164(1–2):69–80. 26724866 10.1016/j.cell.2015.12.017 PMC4715682

[150] Gupta RA, Shah N, Wang KC, Kim J, Horlings HM, Wong DJ, et al. Long non-coding RNA HOTAIR reprograms chromatin state to promote cancer metastasis. Nature. 2010;464(7291):1071–6. 20393566 10.1038/nature08975 PMC3049919

[151] Tsai MC, Manor O, Wan Y, Mosammaparast N, Wang JK, Lan F, et al. Long noncoding RNA as modular scaffold of histone modification complexes. Science. 2010;329(5992):689–93. 20616235 10.1126/science.1192002 PMC2967777

[152] Oh JM, Venters CC, Di C, Pinto AM, Wan L, Younis I, et al. U1 snRNP regulates cancer cell migration and invasion in vitro. Nat Commun. 2020;11(1):1. 31911652 10.1038/s41467-019-13993-7 PMC6946686

[153] Tripathi V, Ellis JD, Shen Z, Song DY, Pan Q, Watt AT, et al. The nuclear-retained noncoding RNA MALAT1 regulates alternative splicing by modulating SR splicing factor phosphorylation. Mol Cell. 2010;39(6):925–38. 20797886 10.1016/j.molcel.2010.08.011 PMC4158944

[154] Chen L, Li Y, Lin CH, Chan TH, Chow RK, Song Y, et al. Recoding RNA editing of AZIN1 predisposes to hepatocellular carcinoma. Nat Med. 2013;19(2):209–16. 23291631 10.1038/nm.3043 PMC3783260

[155] Achour C, Bhattarai DP, Groza P, Roman AC, Aguilo F. METTL3 regulates breast cancer-associated alternative splicing switches. Oncogene. 2023;42(12):911–25. 36725888 10.1038/s41388-023-02602-z PMC10020087

[156] Xiao W, Adhikari S, Dahal U, Chen YS, Hao YJ, Sun BF, et al. Nuclear m(6)a reader YTHDC1 regulates mRNA splicing. Mol Cell. 2016;61(4):507–19. 26876937 10.1016/j.molcel.2016.01.012

[157] Schlesinger D, Elsasser SJ. Revisiting sORFs: overcoming challenges to identify and characterize functional microproteins. FEBS J. 2022;289(1):53–74. 33595896 10.1111/febs.15769

[158] Atanasoai I, Papavasileiou S, Preiß N, Kutter C. Large-scale identification of RBP-RNA interactions by RAPseq refines essentials of post-transcriptional gene regulation. bioRxiv. 2021. 10.1093/nar/gkag090 PMC12956339 41755635

[159] Sekar V, Marmol-Sanchez E, Kalogeropoulos P, Stanicek L, Sagredo EA, Widmark A et al. Detection of transcriptome-wide microRNA-target interactions in single cells with agoTRIBE. Nat Biotechnol. 2024;42(8):1296-302. 10.1038/s41587-023-01951-0 PMC11324520 37735263

[160] Wolin E, Guo JK, Blanco MR, Perez AA, Goronzy IN, Abdou AA et al. SPIDR: a highly multiplexed method for mapping RNA-protein interactions uncovers a potential mechanism for selective translational suppression upon cellular stress. bioRxiv. 2023. 10.1016/j.cell.2025.06.042 PMC12396178 40701149

[161] Jumper J, Evans R, Pritzel A, Green T, Figurnov M, Ronneberger O, et al. Highly accurate protein structure prediction with AlphaFold. Nature. 2021;596(7873):583–9. 34265844 10.1038/s41586-021-03819-2 PMC8371605

[162] Schneider B, Sweeney BA, Bateman A, Cerny J, Zok T, Szachniuk M. When will RNA get its AlphaFold moment? Nucleic Acids Res. 2023;51(18):9522–32. 37702120 10.1093/nar/gkad726 PMC10570031

[163] Wei J, Chen S, Zong L, Gao X, Li Y. Protein-RNA interaction prediction with deep learning: structure matters. Brief Bioinform. 2022;23(1). 10.1093/bib/bbab540 PMC8790951 34929730

